# Overexpressed P75CUX1 promotes EMT in glioma infiltration by activating β-catenin

**DOI:** 10.1038/s41419-021-03424-1

**Published:** 2021-02-04

**Authors:** Anqi Xu, Xizhao Wang, Jie Luo, Mingfeng Zhou, Renhui Yi, Tengyue Huang, Jie Lin, Zhiyong Wu, Cheng Xie, Shengfeng Ding, Yu Zeng, Ye Song

**Affiliations:** 1 Department of Neurosurgery, Nanfang Hospital, Southern Medical University, Guangzhou, Guangdong 510515 PR China; 2 Department of Neurosurgery, The First Hospital of Quanzhou Affiliated to Fujian Medical University, Quanzhou, Fujian, 362000 PR China; 3 Department of Neurosurgery, The First Affiliated Hospital of Gannan Medical University, Ganzhou, Jiangxi 341000 PR China; 4 Department of Neurosurgery, The Second Affiliated Hospital of the Chinese University of Hong Kong (Shenzhen), Shenzhen, Guangdong 518116 PR China; 5 Department of Cell Biology, School of Basic Medical Science, Southern Medical University, Guangzhou, Guangdong 510515 PR China

**Keywords:** CNS cancer, Single-molecule biophysics

## Abstract

The homeobox protein cut-like 1 (CUX1) comprises three isoforms and has been shown to be involved in the development of various types of malignancies. However, the expression and role of the CUX1 isoforms in glioma remain unclear. Herein, we first identified that P75CUX1 isoform exhibited consistent expression among three isoforms in glioma with specifically designed antibodies to identify all CUX1 isoforms. Moreover, a significantly higher expression of P75CUX1 was found in glioma compared with non-tumor brain (NB) tissues, analyzed with western blot and immunohistochemistry, and the expression level of P75CUX1 was positively associated with tumor grade. In addition, Kaplan–Meier survival analysis indicated that P75CUX1 could serve as an independent prognostic indicator to identify glioma patients with poor overall survival. Furthermore, CUX1 knockdown suppressed migration and invasion of glioma cells both in vitro and in vivo. Mechanistically, this study found that P75CUX1 regulated epithelial–mesenchymal transition (EMT) process mediated via β-catenin, and CUX1/β-catenin/EMT is a novel signaling cascade mediating the infiltration of glioma. Besides, CUX1 was verified to promote the progression of glioma via multiple other signaling pathways, such as Hippo and PI3K/AKT. In conclusion, we suggested that P75CUX1 could serve as a potential prognostic indicator as well as a novel treatment target in malignant glioma.

## Introduction

Glioma represents the most frequent and aggressive primary neoplasm within the central nervous system (CNS)^1^. At present, the standard regimen for patients with glioma includes surgical resection, followed by radiation therapy and chemotherapy with temozolomide; however, despite these treatment modalities, the prognosis of patients with glioma remains poor^2–4^. The highly infiltrative form, GBM (grade-IV glioma), exhibits median overall survival (OS) of merely 12–15 months with a 5-year survival rate of <5%^5,6^. The integrated histological and molecular classification system recommended by the 2016 World Health Organization (WHO) Classification of Tumors of the CNS for glioma diagnosis enables more precise patient stratification^7^. Previously, multiple molecular markers, including isocitrate dehydrogenase 1 (IDH1), O-6-methylguanine-DNA methyltransferase (MGMT), and epidermal growth factor receptor, have been used to assess prognosis and treatment response^8,9^. However, it still does not reach the stage that molecular biomarkers cooperate with targeted therapy for precisely individual management. Therefore, further studies are needed to identify the precise molecular mechanism in glioma tumorigenesis, progression, and recurrence to provide valuable prognostic biomarkers and therapeutic targets^10,11^.

In glioma, epithelial–mesenchymal transition (EMT) has an essential role in diffuse infiltrating phenotype and frequently associated with glioma recurrence and malignant development^5,6,12–14^. EMT is an important biological process for epithelial-derived malignant tumor cells to acquire the ability to migrate and invade^15^. It provides tumor cells the infiltrating phenotype characterized by cadherins regulation, cytoskeleton reorganization, special transcription factors (TFs) overexpression, and metabolic change^16^. Accumulating evidences have suggested that a highly sensitive molecular regulator of EMT can be the promising marker of diffuse infiltrating glioma^6,12–14^.

CUX1 (CUT-like homeobox 1 or Cutl1, also known as CCAAT displacement protein, CDP) belongs to the family of homeobox TFs involved in the regulation of proliferation and differentiation^17,18^. *CUX1* gene is located on chromosome 7q22.1 and has been characterized genetically as a haplo-insufficient tumor-suppressor gene with a high frequency of intracellular transcription^17^. Accumulative studies have suggested that CUX1 is widely expressed in human tissues and has an important role in organ development and cell differentiation^19–21^. It is noteworthy that though it is highly expressed in neurons, it remains under-expressed in glial cells^22^. At least three CUX1 protein isoforms have been described: P200, P110, and P75. Recently, studies have highlighted that P110 and P75, the shorter isoforms of CUX1, bind stably to DNA and function as important TFs to promote proliferation, migration, and invasion in various cancer types, including breast cancer^23–26^, prostate cancer^26^, lung cancer^27,28^, pancreatic cancer^29,30^ and pancreatic neuroendocrine neoplasms^31^. However, isoforms expression status of CUX1 and its association with EMT in glioma remains uninvestigated.

In this study, we first identified that the expression of P75CUX1 was mostly expressed among all isoforms in glioma. Also, P75CUX1 was significantly upregulated and associated with poor prognosis of glioma patients. Furthermore, knockdown of CUX1 using siCUX1 or shCUX1 showed inhibition of migration, and invasion of glioma both in vitro and in vivo. Our results highlighted a highly dependent causal relationship between P75CUX1, β-catenin, and EMT. P75CUX1 was found to regulate β-catenin in two ways. First, P75CUX1 promoted β-catenin accumulation in the nucleus; second, CUX1 induced overexpression of β-catenin. Besides, we also verified that CUX1 might regulate several other signaling pathways in glioma, such as Hippo and PI3K/AKT.

## Materials and methods

### Clinical tissue sample collection

A total of 90 paraffin-embedded gliomas and 22 non-tumor brain (NB) tissue specimens were obtained from the Nanfang Hospital of Southern Medical University, Guangzhou, China. All the glioma cases were histopathologically confirmed and classified according to the WHO diagnostic criteria. The patients included 52 men and 38 women with a median age of 38 years (ranging from 3 to 77 years; Table 2). This study was approved by the Ethics Committees of Nanfang Hospital, Guangdong, and written informed consent was obtained from each patient.

### Cell culture

The human glioma cell lines U87 and U251 were purchased from the Chinese Academy of Sciences (Shanghai, China). All cell lines were cultured in Dulbecco’s modified Eagle’s medium (DMEM) (Hyclone, USA) supplemented with 10% fetal bovine serum (FBS, Hyclone, USA) and incubated in a humidified atmosphere of 5% CO_2_ at 37 °C.

### Bioinformatical analyses

Data used in this study for bioinformatical analysis was extracted from online public databases, including Gene Expression Omnibus (GEO; https://www.ncbi.Nlm. nih.gov/gds/), The Cancer Genome Atlas (TCGA; https://cancergenome.nih.gov/), Chinese Glioma Genome Atlas (CGGA; http://www.cgga.org.cn/), and Genotype-Tissue Expression (GTEx; http://commonfund.nih.gov/GTEx/) databases. All the data for each variable were normalized before statistical analyses.

### siRNA transfection

CUX1 knockdown was performed by transfecting cells with specific siCUX1 (5′-CAGAAUUAUUUGACCUAATTUUCGGUCAAAUAAUUCUGTT-3′) targeting human CUX1, designed and synthesized by Genepharma (Shanghai, China). The indicated siRNA was then transfected into cells using Lipofectamine 2000 (Invitrogen, USA) according to the manufacturer’s instructions. The efficiency of siCUX1 was identified with WB assay after 48 h.

### Lentiviral transfection

All lentiviral vectors for CUX1 knockdown with target sequence (5′-CCGGCAGAATTATTTGACCTGAACTCGAGTTCAGGTCAAATAATTCTGTTTTTG-3′) were designed and synthesized by Ribo (Guangzhou, China) using the pLVTHM-GFP lentiviral RNAi expression system. U87 and U251 cells were infected according to the manufacture’s protocol. Polyclonal cells with green fluorescent protein signal were selected for further experiments.

### Plasmid transfection

β-catenin overexpressing plasmid (pcDNA-CTNNB1) and empty plasmid were synthesized from GenePharma (Shanghai, China). Cells were washed with Opti-MEM medium, transfected with Lipofectamine 2000 reagent, and collected for the determination of transfection efficiency at 48 h post transfection.

### Cell proliferation assays

The cell proliferation was determined with Cell Counting Kit-8 (Ribobio, China), according to the manufacturer’s instruction. In brief, cells from different groups were seeded into 96-well multi plates at a density of 1 × 10^3^ cells per well in 100 μL DMEM with 10% FBS. Following incubation for 24 h, a 10 μL CCK8 solution in 100 μl DMEM was added to each well of the plate and further incubated for 1 h. Then, the absorbance was measured using a microplate reader at 450 nm. The cell viability was calculated by the mean of the optical density values in five repeat wells.

### 5-Ethynyl-2ʹ-deoxyuridine (EdU) assay

EdU assay was performed to visualize proliferating cells using Cell-Light EdU Apollo488 in vitro imaging kit (RiboBio, China) according to the manufacturer’s protocol. In brief, differently treated cells were seeded in 96-well plates at a density of 1 × 10^3^ cells/well and incubated for 48 h in triplicate. Then,10 μM EdU was added to the culture medium, and cells incubated for 2 h. Subsequently, cells were fixed with 4% paraformaldehyde and permeabilizated with 0.5% Triton X-100, and cells were stained with the EdU kit. Cell nuclei were stained with 5 μg/ml DAPI for 5 min. The number of EdU-positive cells was counted under a fluorescent microscope (Olympus, USA) in five random fields (×200). Proliferating cells were calculated as the number of EdU-positive cells/the number of DAPI-stained cells. All assays were independently performed in triplicate.

### Cell migration and invasion assay

The migration of U87 and U251 cells were assessed by the wound-healing and Transwell assays. For the wound-healing assay, cells with different treatments were cultured in six-well plates until >90% confluence. Then, cell layers were carefully wounded using a 10 μL sterile pipette tip, and images of wound widths were captured and quantified at 0, 8 h, and 48 h.

Transwell and Boyden assays were performed according to our previous study^32^. Transwell assay was carried out with a Transwell chamber (Costar, USA). Differently treated cells were resuspended in serum-free DMEM at a density of 5 × 10^4^ cells/100 μl and added into upper chamber. The lower chamber was filled with 600 μl DMEM medium supplemented with 10% FBS as a chemoattractant. U87 cells were incubated for 6 h, and U251 cells were incubated for half past 8 h. After incubation, cells that had invaded through the membrane were fixed with 4% paraformaldehyde for 30 min. Cells on the surface outside of the chamber were stained with 1% crystal violet solution for 3 min. Cells were counted under a microscope in five random fields (×200). Boyden assay was carried out with Transwell chambers precoated with 24 mg/ml Matrigel (R&D Systems, USA) for 1 h. U87 and U251 cells were incubated for 24 h, then processed and observed like Transwell assay. All assays were independently performed in triplicate.

### Intracranial xenograft

The animals were cared for in accordance with the Guide for the care and use of laboratory animals in China. All experimental procedures were approved by the Animal Care and Use Committee of the Southern Medical University, Guangzhou, China. Female athymic BALB/c nude mice (3 weeks old) were procured from Sun Yat-sen University (Guangzhou, China). Mice were acclimatized for 1 weeks. Then, mice were anesthetized with 1% pentobarbital sodium and placed in a stereotactic frame (RWD Life Science, China) and injected with U87 cells. A total of 1 × 10^6^ U87 cells transfected with shCUX1 or shCtrl (*n* = 5 per group) suspended in 5 μL PBS were injected into the left hemisphere using a 5 μL syringe and needle (Shanghai Jiaan analyzer factory, China). The site of injection was positioned 1 mm posterior of bregma, 2 mm left of the midline. Mice were killed, and the brains were surgically harvested for Hematoxylin and Eosin (HE) and immunohistochemistry (IHC) staining after 20 days.

### Quantitative real-time PCR (qRT-PCR)

Total RNA was extracted from U251 and U87 cell lines using Trizol (Takara, Japan) and cDNA was synthesized using the cDNA first-strand synthesis kit (Takara, Japan) following the manufacturer’s protocols. QRT-PCR was performed to measure gene expression with the Takara SYBR Green I Real-Time PCR Kit following the manufacturer’s instructions (Takara, Shiga, Japan). Following primers were used for CUX1, forward primer: 5′-CGCCAAAAACAGCACACTCA-3′; reverse primer: 5′-CCGACTTTCAGGCTGGTCTT-3′; for β-catenin, the forward primer was 5′-GGAGGAAGGTCTGAGGAGCA-3′ and reverse primer was 5′-CCAGTGACTAACAGCCGCTT-3′. For GAPDH, the forward primer was 5′-ATCTGTTTCACAGTCTGGGAC-3′, and the reverse primer was 5′-CCTGCTTGTTGGCAAATACC-3′. The qRT-PCR cycle profile was performed at 95 °C for 10 min to activate DNA polymerase, followed by 45 cycles of denaturation at 95 °C for 15 s, annealing at 60 °C for 15 s, and extension at 72 °C for 10 s. The GAPDH was used as an internal reference, and the relative gene expression as fold change was calculated using the 2^−ΔΔCT^ method. Each experiment was repeated three times, and data were presented as mean ± SD from three independent experiments.

### Western Blot

Western blot (WB) was performed according to our previous study^32^. In brief, the total proteins were extracted from cells and tumor tissues with ice-cold RIPA buffer supplemented with 1% protease inhibitors cocktail and 1% phosphoric-acid protease inhibitor at 4 °C for 30 min. Protein concentrations were quantified using the using BCA assay (Beyotime Inc, China). For WB assay, proteins were resolved with 10% sodium dodecyl sulfate-polyacrylamide gel electrophoresis followed by transfer onto a polyvinylidene difluoride membranes (Invitrogen, Carlsbad, CA). Subsequently, the membranes were blocked with 5% BSA in Tris-buffered saline with Tween-20 (TBST; pH 7.0) for 1 h and probed with primary antibodies overnight at 4 °C. The primary antibodies included CUX1 (Proteintech, 11733-1-AP, 1:1000), E-cadherin, N-cadherin, Snail, Slug, Claudin, β-catenin, ZO-1 (Cell Signaling Technology, EMT Kit, 1:1000), phospho-β-cateninSer33/37/Thr41 (#9561; Cell Signaling Technology), GAPDH, β-actin (1:1000; Cell Signaling Technology) antibodies. The secondary antibodies included anti-rabbit or anti-mouse IgG antibody (1:2000; CoWin Bioscience, Beijing, China). Subsequently, the membranes were washed five times with TBST and incubated with horseradish peroxidase-conjugated secondary antibodies for 1 h at room temperature. The target bands were visualized using the enhanced chemiluminescence Kit, and the protein bands were quantified using ECL (Pierce, USA). GADPH and β-actin were used as an internal control. Each experiment was repeated three times, and data were presented as mean ± SD from three independent experiments.

### IHC and staining evaluation

IHC was performed according to our previous study^32^. In brief, 4 μm paraffin sections were deparaffinized in 100% xylene and rehydrated in an ethanol gradient. Antigen retrieval was performed by heating the tissue sections at 100 °C in citrate buffer for 15 min. Moreover, endogenous peroxidase activity and non-specific antigens were blocked by incubation with peroxidase blocking reagent containing 3% hydrogen peroxide and serum for 5 min. Subsequently, the sections were incubated with primary antibodies overnight at 4 °C. The primary antibodies used were as follows: CUX1(Proteintech, 11733-1-AP, 1:100), E-cadherin, N-cadherin, Snail, Slug, β-catenin (Cell Signaling Technology, EMT Kit, 1:100). The next day, the sections were washed with PBS three times for 5 min gently and incubated with biotin-labeled secondary antibody for 1 h at room temperature and washed three times with PBS. The immunostaining was carried out by staining with 3, 3′-diaminobenzidine chromogen (GT Vision II IHC Detection Kit, Gene Tech, Shanghai, China) counter-stained with hematoxylin, dehydrated, and mounted and the sections were examined under a bright-field microscope equipped with a digital camera (Nikon, Japan). The positive proportion of stained tumor cells was scored as follows: ≤5% positive cells (score of 0), 6–25% positive cells (score of 1), 26–50% positive cells (score of 2), ≥51% positive cells (score of 3). For the statistical analysis of nuclear expression, the score of 0–2 was considered to be a low expression, and the score of 3 was considered to be a high expression. All experiments were independently performed in triplicate.

### Immunofluorescence (IF)

All antibodies used were as follows: CUX1, β-catenin (both Proteintech, 1:100). U251 and U87 cells were seeded on coverslips and cultured for over 8 h. Subsequently, cells were fixed in 3.5% paraformaldehyde in PBS and permeabilized in 0.3% Triton X-100 at room temperature. Then, cells were blocked with goat serum and incubated with primary antibodies in 4 °C overnight. After washing with PBS three times, cells were incubated with secondary antibodies 1 h at room temperature. Subsequently, the nuclei were stained with 4′,6-diamidino-2-phenylindole (0.2 mg/ml DAPI) for 10 min prior to imaging. Then, cells were washed with PBS, sealed with Fluoromount-G, and observed under the fluorescent microscope.

### TOP‐Flash/FOP‐Flash reporter assay

TOP/FOP assay was performed according to our previous study^33^. In all, 5 × 10^4^ cells with shCUX1 or shCtrl were, respectively, seeded onto a 24‐well plate. TOP‐Flash reporter plasmids were transiently transfected into the cells with Lipofectamine 2000 Invitrogen. After transfection for 48 h, a Dual‐Luciferase Assay Kit (Promega) was used to detect the activities of both firefly and Renilla luciferase reporters according to the manufacturer’s instructions. TOP‐Flash reporter activity was calculated as the relative ratio of firefly luciferase activity to Renilla luciferase activity. All experiments were performed in triplicate.

### Statistical analysis

All statistical analyses were performed using SPSS 13.0 and Graph Pad Prism 7.0. Data were expressed as mean ± SD of independent experiments unless indicated otherwise. Two-tailed Student’s *t* test was used to analyze the differences in the results between the two groups. Comparisons among three or more groups were assessed using one-way analysis of variance. Survival analysis was performed using the Kaplan–Meier method and was compared with the log-rank test. Correlation between CUX1 and Ctnnb1 expression was analyzed by the Spearman correlation method. A Chi-square test or Fischer’s exact test was used to identify the differences between categorical variables. All tests were two-tailed, and *p* < 0.05 was considered statistically significant.

## Results

### Expression of *CUX1* in glioma

**Fig. 1:**
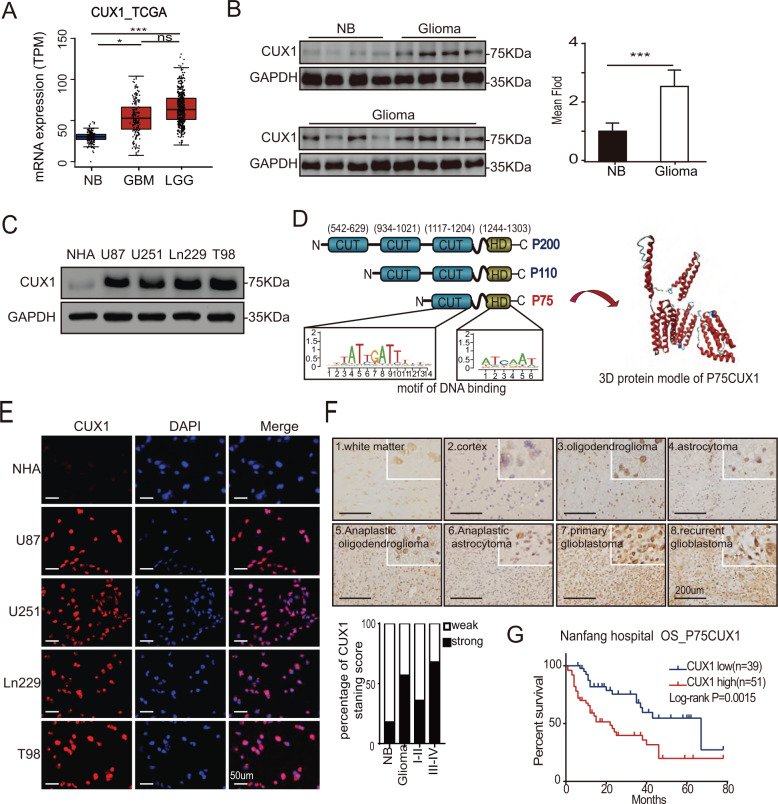
P75CUX1 expression in NB and glioma cells. **a** The expression level of CUX1 mRNA was increased in glioma compared with NB tissues (GBM, *n* = 163; LGG, *n* = 518; NB, *n* = 207,). CUX1 mRNA expression data was derived from RNA sequencing array of TCGA and GTEx databases, analyzed with the GEPIA webserver (*P < 0.05, ***: *P* < 0.001). **b** P75CUX1 protein expression was evaluated in 12 glioma tissue samples compared with four normal tissue samples with WB assay (***:*P* < 0.001). **c** P75CUX1 protein expression of CUX1 in normal glial cell and glioma cell lines was examined with WB assay. **d** Domain Organization of P75CUX1 and function of the Domains. *CUT* CUT domain, *HD* Homeobox DNA-binding domain and the CUT domain is DNA-binding and protein-interacting motif, which can bind independently or in cooperation with the homeodomain. And HD domain binds DNA through a helix-turn-helix type of structure. The motif of binding DNA in the domain was analyzed with Cristome. 3D model of protein was designed with I-TASSER (C-score = −0.79, Estimated TM-score = 0.61 ± 0.14). **e** P75CUX1 expression and localization of CUX1 in normal glial cell lines and glioma cell lines were examined by IF assay. **f** P75CUX1 expression in NB tissues and glioma was examined by IHC staining (NB, *n* = 90; glioma, *n* = 22), including normal white matter (1), normal cortex (2), oligodendroglioma (grade-II) (3), astrocytoma (grade-II) (4), anaplastic oligodendroglioma (grade-III) (5), anaplastic astrocytoma (grade-III) (6), primary glioblastoma (grade-IV) (7), and recurrent glioblastoma (grade-IV) (8). **g** Kaplan–Meier survival analysis for overall survival of patients with glioma based on protein expression of P75CUX1 (90 glioma samples were collected from Nanfang hospital, *P* = 0.0015).

Analysis of public data sets revealed that CUX1 mRNA expression was overexpressed in glioma, including Low-grade glioma and GBM, as compared to NB (Fig. 1a). As CUX1 mRNA could not fully represent the expression levels of CUX1 isoforms in glioma, we performed the WB assays to distinguish the expression of CUX1 isoforms in glioma (Supplementary Fig. 1). P75CUX1 tested clearly higher expression in glioma tissues than in NB tissues (Fig. 1b), and higher expression in glioma cell lines than in glial cell (Fig. 1c). Organization of protein showed that the P75CUX1 as an important transcription factor (TF) has two DNA-binding motif (Fig. 1d). IF staining showed that P75CUX1 was only expressed in nucleus, and the expression was significantly higher in glioma cells than glial cells (Fig. 1e). IHC staining of P75CUX1 was performed in 90 paraffin-embedded glioma tissue specimens and 22 NB tissues from our department. We observed moderate staining in neurons and week staining in glial cells, respectively, in the cortex and the white matter (Fig. 1f). The analysis also confirmed high protein expression level of P75CUX1 in 56.67% (51 of 90) glioma tissues and only 18.18% (4 of 22) normal tissues (Fig. 1f, Table 1). Besides, the P75CUX1 expression level was significantly positively correlated with the pathological grading, indicating that high-grade tumors had higher expression of P75CUX1 than the low-grade tumors (WHO III-IV vs. WHO I–II) (*P* = 0.003) in patients with glioma (Fig. 1f, Table 2). In conclusion, as a nucleus-located protein, P75CUX1 was overexpressed in glioma, and its expression was positively correlated with tumor grade.

**Table 1:** p75CUX1 protein expression between glioma and non-tumor brain (NB) tissues.

Group	*n*	Protein expression		*P* value
		High expression	Low expression	
NB	22	4	18	
Glioma	90	51	39	0.001

**Table 2:** Association between the clinicopathologic characteristics and the P75CUX1 protein expression in glioma.

Characteristics	*n*	Protein expression (%)		*p* value
		High expression	Low expression	
*Gender*				0.275
Male	52	32(61.5)	20(38.5)	
Female	38	19(50.0)	19(50.0)	
*Age*				0.604
<50	74	41(55.4)	33(44.6)	
≥50	16	10(62.5)	6(37.5)	
*Histologic type*				0.883
Astrocytic tumors	61	34(55.7)	27(44.3)	
Oligodendrogial tumors	18	10(55.6)	8(44.4)	
Other	11	7(63.6)	4(36.4)	
*WHO grade*				0.003
I + II	31	11(35.5)	20(64.5)	
III + IV	59	40(67.8)	19(32.2)	

### P75CUX1 overexpression predicted poor survival of patients with glioma

The analysis of survival data obtained from the medical records of the patients with glioma from Nanfang hospital revealed that high P75CUX1 expression was significantly correlated with the poor OS of glioma patients (Fig. 1g). In contrast to previous analysis in glioma^34^, our study did not support the prognostic value of CUX1 mRNA. Although the CUX1 mRNA expression in GBM was positively correlated with the prognosis as analyzed using TCGA_GBM data, no supportive evidence could establish the role of CUX1 mRNA as a prognostic indicator in GBM or glioma after combining CGGA and TCGA data (Supplementary Fig. 2).

### P75CUX1 knockdown inhibited cell migration and invasion but not proliferation in vitro

**Fig. 2:**
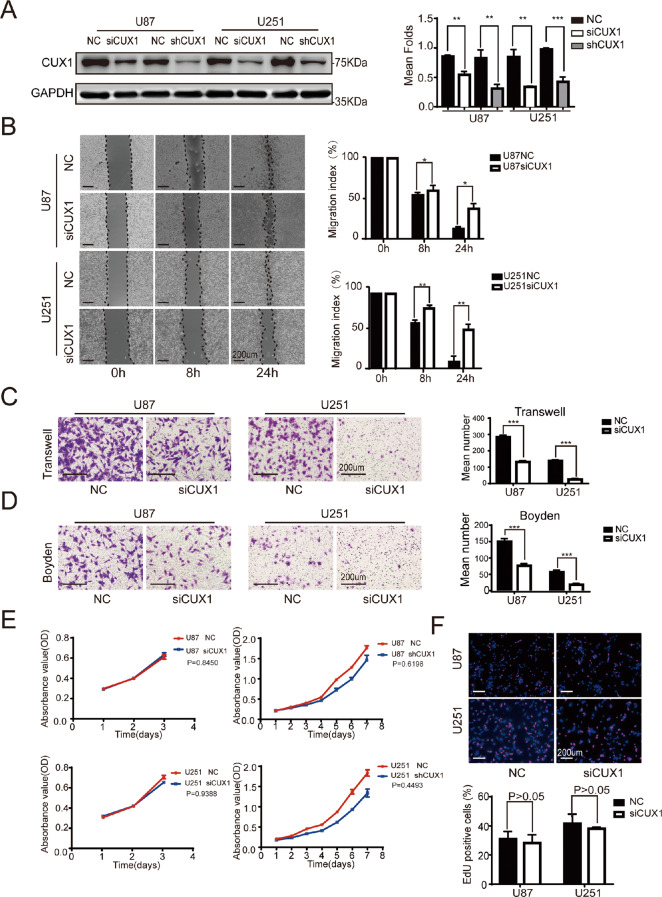
CUX1 knockdown inhibited cell migration and invasion but not cell proliferation in vitro. **a** siRNA and shRNA against CUX1 decreased the expression of P75CUX1 in U87 and U251 cells compared with control cells by WB assay (**:*P* < 0.01; ***:*P* < 0.001). **b** Wound-healing assays indicated the siCUX1 transfection impaired cell migratory capacity, compared with the NC group (*P < 0.05; **P < 0.01). **c** Transwell chamber assays revealed that CUX1 knockdown with siRNA transfection reduced the migratory capability of U87 and U251 cells (***P < 0.001). **d** Boyden chamber assays showed that CUX1 knockdown with siRNA transfection reduced the invasive ability of U87 and U251 cells (***P < 0.001). **e** CCK8 assays measured the modest effect on the proliferation of U87 and U251 cells following downregulation of CUX1 expression. **f** EDU incorporation assays revealed that the percentage of cells in the S phase had no statistical significance following downregulation of CUX1 (*P* > 0.05).

siRNA and shRNA vectors were used to transiently and stably knockdown P75CUX1 expression in U251 and U87 cells. The efficiency was confirmed with the WB assay (Fig. 2a). Subsequently, we examined the effect of P75CUX1 knockdown on the migration and invasion abilities of glioma cells in vitro. Wound-healing assays indicated a significant reduction in wound closure area of P75CUX1 knockdown cells at 24 h compared with negative control (NC) cells (Fig. 2b). Consistent results were also observed in the Transwell assays, P75CUX1 knockdown significantly reduced the percentage of migrated cells (Fig. 2c). In the Boyden assays, cells with P75CUX1 knockdown exhibited decreased invasion capacity compared with NC cells (Fig. 2d).

We also assessed the effect of P75CUX1 knockdown on the proliferation ability of glioma cells in vitro. The CCK8 assays revealed that the P75CUX1 knockdown did not inhibit cell proliferation (Fig. 2e). Similar results were observed with EdU assays (Fig. 2f).

### P75CUX1 knockdown inhibited cell migration and invasion in in vivo

**Fig. 3:**
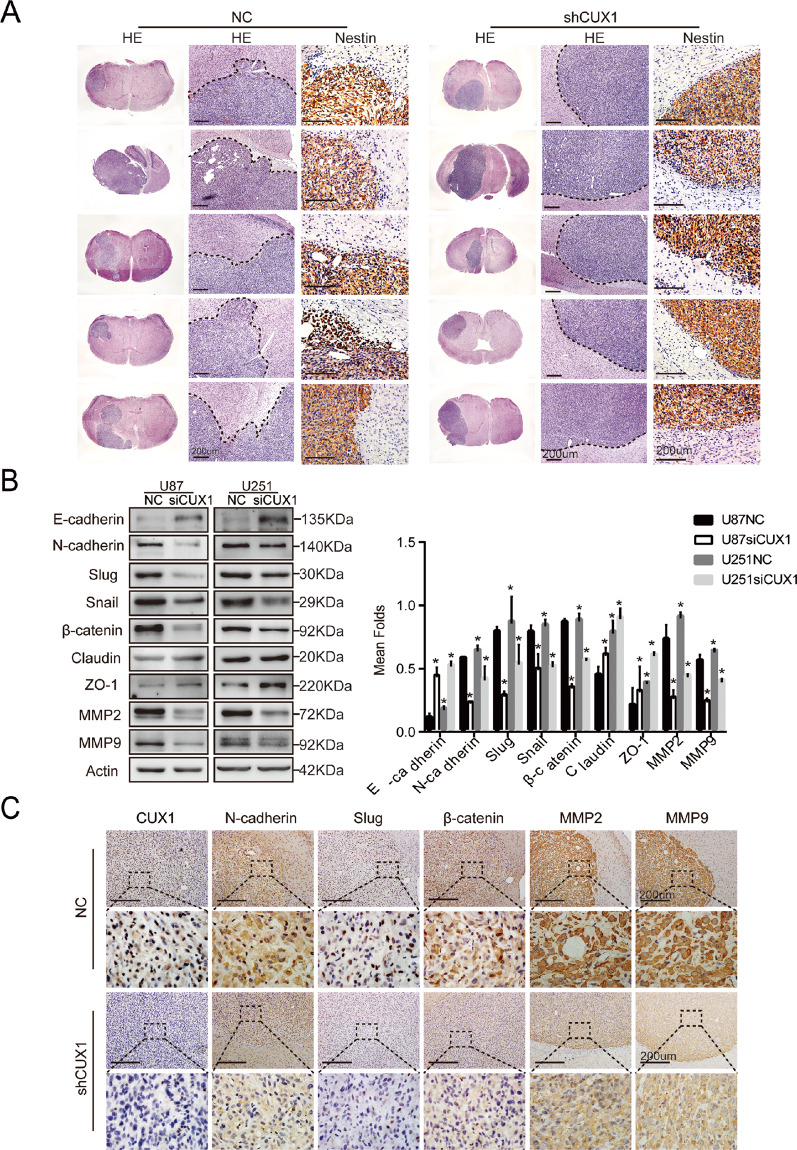
CUX1 knockdown inhibited cell migration and invasion by regulating EMT progression and MMPs expression in vitro and in vivo. **a** Representative images of intracranial tumor xenograft with U87 cell lines in nude mice. HE and IHC of human Nestin staining revealed that the CUX1 knockdown inhibited the aggressive phenotype in vivo (NC, *n* = 5; ShCUX1, *n* = 5). **b** WB assay revealed that the CUX1 knockdown in U87 and U251 cells regulated the expression of EMT-associated proteins and MMPs in vitro, including reduced the expression of N-Cadherin, Slug, Snail, β-Catenin, MMP2, and MMP9, and enhanced the expression of E-Cadherin, Claudin, and ZO-1(*P < 0.05). **c** IHC staining showed that CUX1 knockdown inhibited the EMT-related protein expression including N-Cadherin, Slug, β-catenin, MMP2, and MMP9 in vivo.

Next, we further validated the effect of P75CUX1 on migration and invasion in glioma xenograft model in vivo. U87 cells infected with shCUX1 or shCtrl, respectively, were administered into the brain of nude mice (shCUX1, *n* = 5; shCtrl (NC), *n* = 5). The NC group formed tumor tissues with more significant invasion and ill-defined margins and multiple protrusions toward the normal brain tissue, whereas the shCUX1 group formed tumor tissues with relatively smooth and clear edges with the normal brain tissue, revealed by HE and IHC of human Nestin (Fig. 3a). Thus, the above findings indicated that CUX1 promoted the infiltrative phenotype of glioma in vitro and in vivo.

### P75CUX1 knockdown suppressed EMT and MMPs expression

To determine the underlying molecular mechanisms of P75CUX1 on the infiltrative phenotype of glioma, the expression levels of EMT-associated proteins and MMPs were examined both in vivo and in vitro. Significantly elevated expression of E-cadherin, Claudin, ZO-1, and reduced expression of N-cadherin, slug, snail, β-catenin, MMP2, MMP9 were detected in U87 and U251 cells with P75CUX1 knockdown in vitro (Fig. 3b). Moreover, P75CUX1 knockdown significantly downregulated EMT-associated proteins expression, including N-cadherin, Slug, β-catenin, and MMPs, confirmed by IHC results of intracranial tumor xenograft models (Fig. 3c).

### P75CUX1 regulated β-catenin expression and activity in glioma

**Fig. 4:**
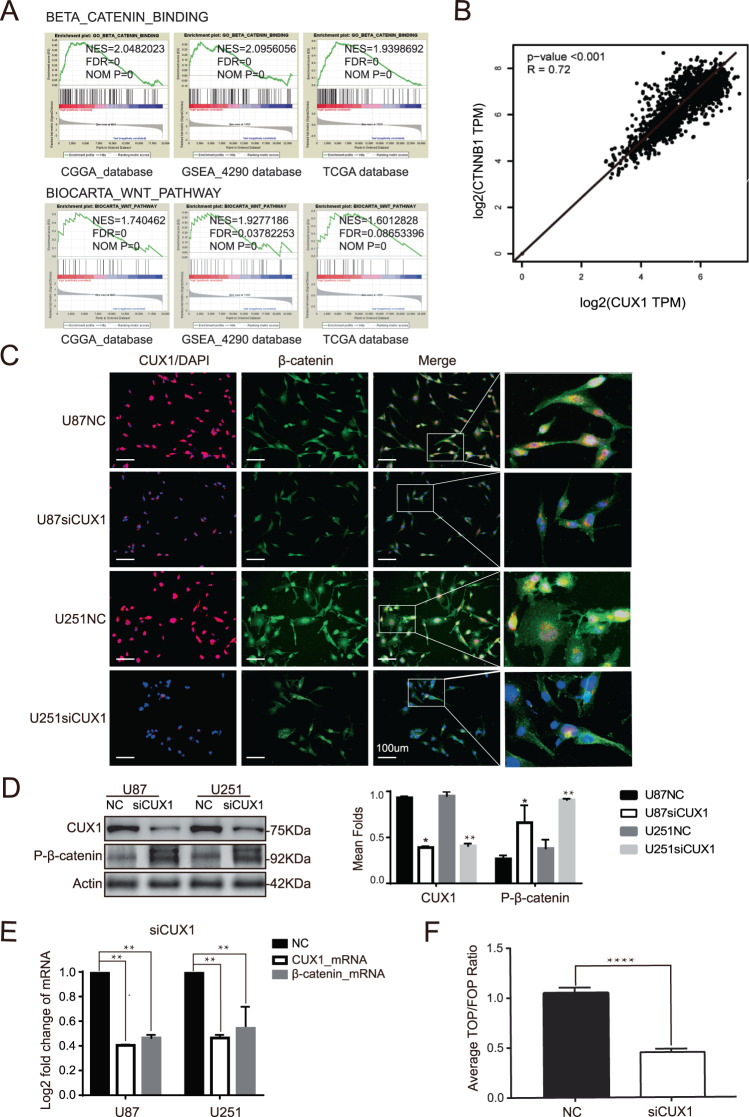
P75CUX1 regulated β-catenin expression and activated WNT/β-catenin signaling pathway. **a** Gene set enrichment analysis (GSEA) showed that there was a significant correlation between CUX1 mRNA expression and the WNT/β-catenin pathway in glioma (data from TCGA, CGGA, and GSE4290 databases). **b** Spearman correlation analysis indicated a significant correlation between the mRNA expression of CUX1 and β-catenin in glioma and NB tissues (*P* < 0.001, *R* = 0.72, data from TCGA and GTX database). **c** IF assay revealed that CUX1 knockdown inhibited the total β-catenin expression and β-catenin accumulation in the nucleus of U87 and U251 cells. **d** WB showed that P-β-catenin (Ser33/37/Thr41) expression increased while P75CUX1 knockdown. **e** qRT-PCR analysis showed that the CUX1 knockdown reduced the mRNA expression of β-catenin in U87 and U251 cells (**P < 0.01). **f** TOP/FOP flash assay detected that the CUX1 knockdown suppressed the activity of Wnt/β-catenin in the nucleus. Bars indicated the relative luciferase activity (****P < 0.0001).

Through gene set enrichment analysis (GSEA), we identified and predicted the possible biological function of CUX1 in glioma. An evident correlation of CUX1 mRNA expression with the Wnt/β-catenin pathway and β-catenin binding activity was found (Fig. 4a). Furthermore, we also observed a significant positive correlation between CUX1 and β-catenin mRNA expression levels by analyzing the mRNA data of glioma and NB tissues from TCGA and GTEx databases using GEPIA webserver (Fig. 4b). Both WB findings on cell lines and IHC data from glioma xenograft specimens indicated that the expression of total β-catenin was decreased when CUX1 knockdown, and nuclear accumulation of active β-catenin were also relatively lower (Fig. 3b, c). Consistently, a similar result was also revealed by IF in vitro (Fig. 4c). WB showed that P-β-catenin (Ser33/37/Thr41) expression increased while P75CUX1 knockdown (Fig. 4d). Furthermore, qRT-PCR assays also indicated that P75CUX1 knockdown significantly inhibited β-catenin mRNA expression (Fig. 4e). TOP/FOP flash assay also revealed that the downregulation of CUX1 markedly suppressed the activity of Wnt/β-catenin signaling pathway (Fig. 4f). Collectively, CUX1 regulated the expression of total β-catenin and nuclear activity of β-catenin in glioma.

### P75CUX1 regulated glioma cell EMT, migration, and invasion mediated via β-catenin

**Fig. 5:**
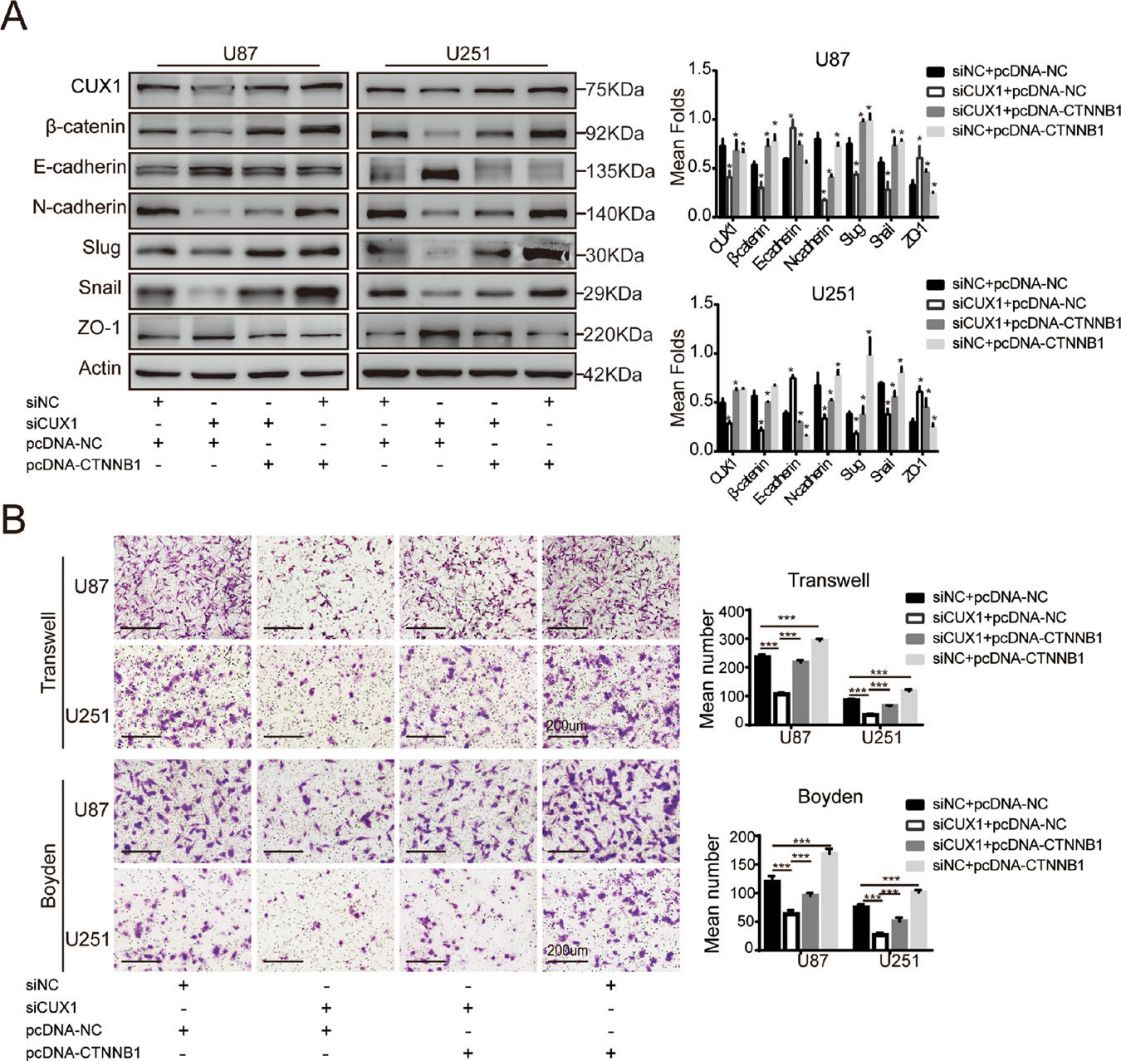
β-catenin mediated the effect of CUX1 on EMT process, migration, and invasion. **a** WB assay showed that β-catenin overexpression could reverse the decreased EMT process induced by siCUX1 in U87 and U251 cells (*P < 0.05). **b** Transwell and Boyden assays indicated that β-catenin overexpression could reverse the decreased migratory and invasive capacity induced by siCUX1 in U87 and U251 cells (***P < 0.0001).

We hypothesized that P75CUX1 promoted EMT process mediated via β-catenin dependent regulation. To prove our assumption, we used CUX1 knockdown U87 and U251 cells transfected with β-catenin plasmid to recover β-catenin expression. The results revealed that EMT markers were moderately recovered with exogenous β-catenin (Fig. 5a). Likewise, the migration and invasion capacities of CUX1 knockdown U87 and U251 cells were both partially recovered by exogenous β-catenin (Fig. 5b). Taken together, the results indicated a highly dependent causal relationship of CUX1, β-catenin, and EMT in glioma progression.

### P75CUX1 affected several signaling pathways in glioma

**Fig. 6:**
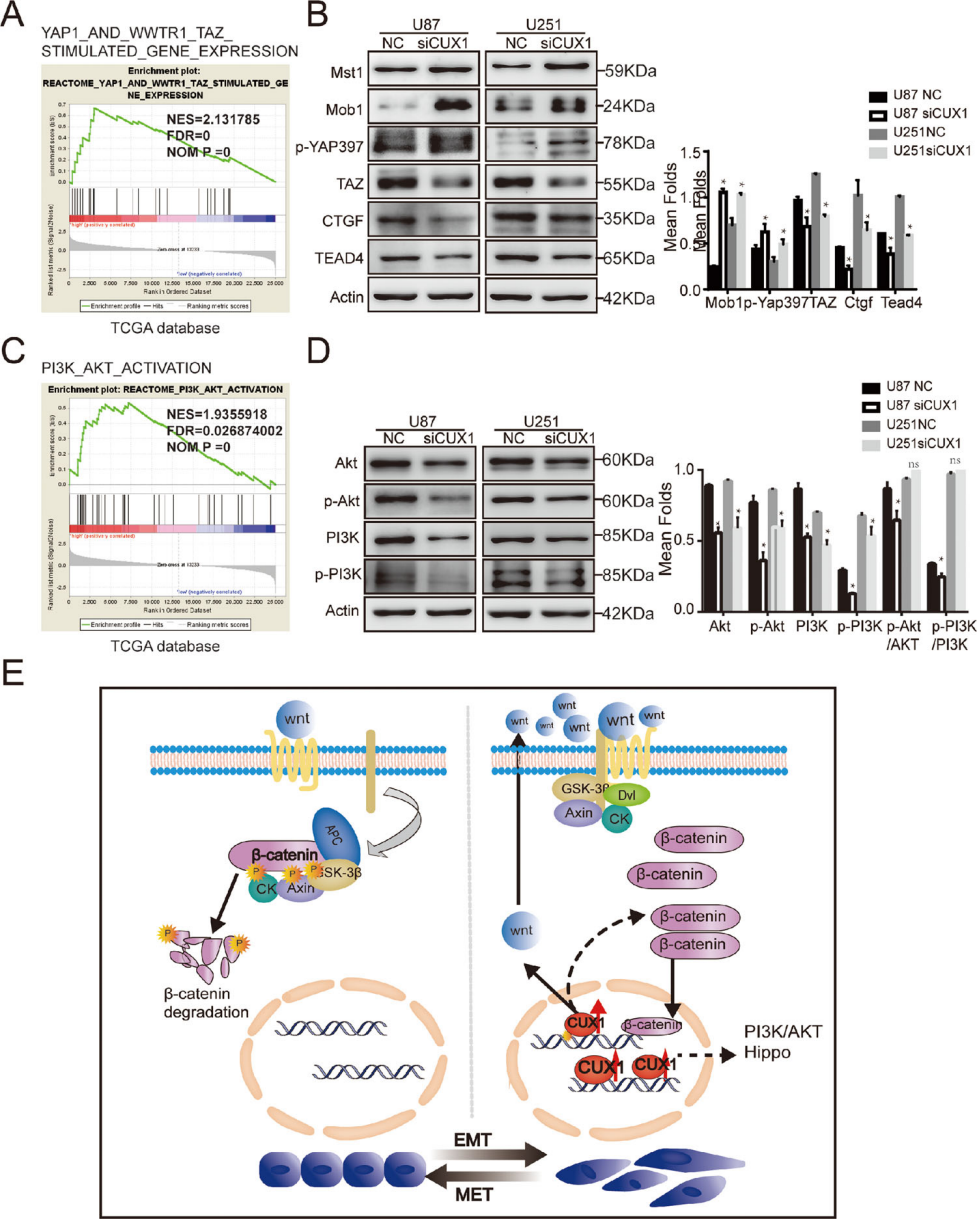
P75CUX1 was also regulated multiple other signaling pathways. **a** GSEA showed that there was a significant correlation between CUX1 and Yap/ TAZ expression, which are the key regulators of Hippo signal pathway in glioma. **b** CUX1 knockdown regulated the expression of proteins in Hippo pathway, including Mst1, Mob1, p-YAP, TAZ, CTGF, and TEAD4. **c** GSEA showed that CUX1 expression was closely associated with PI3K/AKT activation in glioma. **d** CUX1 knockdown inhibited the expression of key proteins and their phosphorylated forms in the PI3K/AKT pathway, including PI3k, AKT, P-PI3K, and P-AKT, but reduced their phosph/total ratio. **e** A schematic representation illustrating the role of P75CUX1 in regulating EMT in glioma cells. Based on the findings of this study, P75CUX1 could activate the Wnt/β-catenin pathway and induce β-catenin expression, which eventually promotes EMT. Furthermore, Wnt/beta-catenin pathway may be activated in ligand-independent manner and non-ligand-independent manner (respectively, showed with solid and dotted line).

Though it was verified that β-catenin is a primary downstream signaling molecule of the P75CUX1-mediated EMT to promote glioma infiltration, we further explored the mechanisms of P75CUX1 regulation in glioma. GSEA results showed that P75CUX1 might regulate the Yap/TAZ expression, which are the key regulators of Hippo signal pathway in glioma with TCGA database. (Fig. 6a). Consistently, significantly decreased expressions of TAZ was observed after P75CUX1 knockdown in vitro. In addition, the expression of several other markers of Hippo pathway, such as CTGF, TEAD4 were decreased, while p-YAP, Mst1, Mob1 were increased (Fig. 6b). GSEA also showed the effect of P75CUX1 on the PI3K/AKT activation (Fig. 6c). WB verified that expression level of AKT, P-AKT, PI3K, and P-PI3K were decreased after P75CUX1 knockdown in vitro, though the phospho/total ratio was not changed (Fig. 6d). Hippo and PI3K/AKT pathway were known as important signal pathway regulating glioma development. Overall, we demonstrated that P75CUX1 could promote EMT process and enhance the invasion and migration capacity of glioma mainly by activating not only the Wnt/β-catenin pathway, but also several other pathways such as Hippo and PI3K/AKT pathways (Fig. 6h).

## Discussion

To date, molecular studies related to protein isoforms as predictive biomarkers of tumor stage and treatment outcomes remain mostly unexplored; nevertheless, previous studies have indicated the significance of improved understanding of the diversity of protein isoforms produced by mutation, alternative splicing, or protein modification as crucial mechanisms of tumor progression^35–37^. Notably, expression, function, and the prognostic relevance of different protein isoforms have been previously found to be associated with glioma phenotype and responses to therapy^35,36,38^. For instance, IDH1 R132H mutation represents the predominant mutation type in glioma and patients with IDH1 R132H mutation exhibit better prognosis than those with IDH1 wildtype^39^. In the present study, we first indicated that P75CUX1 is the most frequently expressed CUX1 protein isoform in glioma, with specifically designed antibodies for WB assays to distinguish all CUX1 isoforms (Supplementary Fig. 1).

The heterogeneity of glioma is a great challenge in the treatment of glioma, and the resistance to chemotherapy and radiotherapy is closely related to this heterogeneity. For example, IDH1-mutation are important markers to show heterogeneity of glioma with different biological characteristics and better prognosis than IDH1-Wildtype glioma^40^. Besides, MGMT promoter methylation was an independent prognostic biomarker of glioma sensitive to temozolomide and radiotherapy^41^. Similarly, we observed that P75CUX1 was heterologously expressed in glioma (Fig. 1 and Table 2). At present, there is no effective treatment for glioma with high heterogeneity. We believe that our study about P75CUX1 is meaningful to explore the individualized glioma treatment regimen to improve the survival rate of patients

*CUX1* gene produced three isoforms: P75CUX1, P110CUX1, and P200CUX1^17,18^. P75CUX1 was generated owing to the loss-of-function somatic mutation at intron 20 to AUG (new starting point of transcription)^17,18,42^. The P110CUX1 isoform is proteolytically processed by cathepsin L to full length form P200CUX1^17,18,43^. P200CUX1 comprises four evolutionally conserved DNA-binding domains, three repetitive CUT domain and a cleavage homologous domain (HD). Although, the NH2 terminal of P200CUX1 contains a self-inhibiting domain that prevents DNA binding^18,44–46^^,^. The P110CUX1 contains two CUT repeats and a cleavage HD, and P75CUX1 contains only one CUT repeat and one cleavage HD^18^. Both P110 and P75 isoforms function as important TFs with stable DNA-binding activity^18,23,47^.

Our study provided the firm evidence of the oncogenic role of P75CUX1 in glioma. Notably, P75CUX1 protein expression was significantly higher in glioma tissue compared with NB tissues. Furthermore, we also observed that P75CUX1 expression level was positively correlated with tumor grade in glioma; however, no significant association was observed between P75CUX1 expression levels and other clinicopathological characteristics, including age, sex, or histologic type in the 90 patients with glioma obtained from Nanfang hospital. Survival analysis indicated that P75CUX1 was a poor prognostic indicator for patients with glioma. Moreover, our results showed knockdown of P75CUX1 markedly inhibited the migration and invasion in glioma both in vitro and in vivo. Considering the main expressed CUX1 isoform in glioma is P75CUX1, our study revealed that targeting P75CUX1 was a potential effective strategy for glioma therapy.

The specific phenomenon that P75CUX1 significantly regulated the migration and invasion but not the proliferation of glioma cells facilitated us to reveal novel mechanisms of rapid infiltrating progression in glioma. The mechanism of correlation of CUX1 and EMT was the breakthrough finding because EMT is a well-recognized mechanism in glioma diffuse infiltration^12–14^. In our study, when P75CUX1 was knocked down, E-cadherin, ZO-1, and Claudin were significantly augmented while N-cadherin, β-catenin, Slug, Snail, MMP2, and MMP9 were reduced in vitro and in vivo, indicating a more epithelial phenotype under P75CUX1 inhibition. By the way, because of the low expression of E-cadherin in glioma tissues, E-ca could be detected with WB in vitro, but not IHC in vivo in this study. In addition, significant affected N-ca was detected by WB and IHC in this study as previous studies showed. N-ca is a relatively reliable indicator of cadherins to show glioma EMT process^48–50^. Supported with several EMT markers including N-ca, our findings suggested that P75CUX1 was a crucial effector molecule that induced EMT in glioma.

Predominantly, β-catenin is translocated into the nucleus and together with lymphoid enhancer factor/T-cell factor factors induces the transactivation of target genes, including cadherins, and other key TFs such as Snail and Slug, which promotes EMT directly and indirectly^13,30,51–53^. GSEA using TCGA, CGGA, and GSE4290 databases revealed close association of CUX1 with the Wnt/β-catenin pathway and β-catenin binding. A significant positive correlation between β-catenin and CUX1 mRNA expression was also observed. Moreover, our research indicated that P75CUX1 regulated β-catenin expression and phosphorylation (Ser33/37/Thr41). TOP/FOP reporter assay finally verified that CUX1 induced β-catenin activity in the nucleus. As the schematic representation illustrated, we proposed a novel molecular mechanism underlying P75CUX1 function in glioma infiltration, that P75CUX1 promoted EMT by inducing β-catenin expression and activating Wnt/β-catenin pathway and (Fig. 6e). As we all know, Gsk3β phosphorylates Ser33/37/Thr41 of β-catenin when WNT signal absent, and then β-catenin is recognized by E3 ubiquitin ligase, labeled ubiquitin, and finally degraded by proteases^54^. In this paper, we demonstrated that P75CUX1 prevented the phosphorylation-dependent degradation of β-catenin and promoted its activity in the nucleus. In breast cancer study, the production of Wnt regulated by CUX1 has been proven^55^. Whether a similar mechanism between CUX1 and WNT exists in glioma need more profound exploration.

Besides CUX1/ β-catenin/EMT axle, to complete the mechanism of P75CUX1 in glioma, our further investigation indicated that P75CUX1 also regulated the expression of several important targets in glioma, including Hippo and PI3K/AKT signaling pathways in glioma. These signal pathways are also known to enhance mesenchymal features of glioma, and have been reported to be regulated by CUX1 in several cancers such as breast cancer^56–58^.

Our study has several limitations that must be acknowledged. First, as P75CUX1 shared the same domain with P110CUX1 and P200CUX1, it was highly difficult just to target P75CUX1 and keep the other isoforms to understand how P75 isoform exactly functions; therefore, validation of mechanism of only the P75CUX1 in glioma warrants further investigation. Second, Wnt/β-catenin signal pathway is well-recognized and closely related with EMT, Hippo, and PI3K/AKT signal pathways in glioma stem cells^13,53,57,59^, thus the mechanism of P75CUX1 in glioma might be more complicated than this study presents.

In summary, the present study demonstrated that P75CUX1 could serve as a poor prognostic indicator in glioma, and CUX1/ β-catenin/EMT axle plays a crucial role in glioma infiltration. We believe that further studies targeting P75CUX1 will provide insight into novel therapeutic strategies to prevent the rapid development of diffuse infiltrative glioma.

## Supplementary information

Supplementary figure legends.

Supplementary file of P75CUX1 target

Supplementary remark of CUX1 antibody

Supplementary Fig. 1

Supplementary Fig. 2
