# Supplementary material for: Overexpressed P75CUX1 promotes EMT in glioma infiltration by activating β-catenin
Source: Cell Death Dis. 2021 Feb 4;12(2):157. doi: 10.1038/s41419-021-03424-1 (PMC7862635; doi:10.1038/s41419-021-03424-1)
Supplement: Supplementary file 1 — Supplementary figure legends. [file 41419_2021_3424_MOESM1_ESM.docx]

**Supplementary Fig. 1 The expression status of CUX1 isoforms in glioma.** **a** In glioma tissues and cell lines, P75CUX1 was the most frequently expressed isoform of CUX1 isoforms, while P200CUX1 and P110CUX1 were relatively absent. **b** P75, P110, P200 CUX1 isoforms could be detected with the antibody in jurkat cell line, which is a kind of T lymphoid cell line.

**Supplementary Fig. 2 Kaplan-Meier survival analysis of overall survival (OS) in glioma patients based on mRNA expression of CUX1 with TCGA and CGGA databases. a** OS analysis of CUX1 mRNA in TCGA_GBM (P=0.0124). **b** OS analysis of CUX1 mRNA in CGGA_GBM (P=0.3931). **c** OS analysis of CUX1 mRNA in TCGA_Glioma (P=0.0011). **d** OS analysis of CUX1 mRNA in CGGA_Glioma (P=0.2441). The log-rank test was used to calculate P values
