# Supplementary material for: Overexpressed P75CUX1 promotes EMT in glioma infiltration by activating β-catenin
Source: Cell Death Dis. 2021 Feb 4;12(2):157. doi: 10.1038/s41419-021-03424-1 (PMC7862635; doi:10.1038/s41419-021-03424-1)
Supplement: Supplementary file 2 — Supplementary file of P75CUX1 target [file 41419_2021_3424_MOESM2_ESM.docx]

-- Input sequence of CTNNB1 promoter 2000Kb---------------------------------------------------

>hg38_ncbiRefSeq_NM_001276352.2 range=chr1:67134971-67136970 5'pad=0 3'pad=0 strand=- repeatMasking=nÐ

GTATTTATCTATAATAGGTTCATGAACTCATTCAGTAAATACTGAATTTAACTGAGAGCAATTAAAGTAGTTAGGAAAAAATCCACTTGAATTCTTTTTTGTGTACATTTACCAAACATTTGTTATTAAAGAGAAAATACTTTGTACTTTGTGTTATATTAATAATTGTATTACTAATTATAAGATGCTGGGCAAATCACATAGTTTCTTTGAATTTCAGTCAGGTCTTTTCTAAAATCAAAGATAATACCCTTTCCATAGATACGAATTAAGGTTTAAGAATGAAAAGTAAAGGATGAAATTAGAACTATTTTAAAGTTGTAGCCTAGTACCTGACATGCAGAACGTGTTCCATAAATATTAACTGAGATAATGATAATATTCTAAGAATTTCATAAGATTCATGCTTTACAAATTTTATTGACTGAAGATTAATGAGTAGCTAACGTTTCTGAACTGAATTCTTAAGAAGCTAATTTTCACCTCTGTACAAATTTTTGAGCTGAAGGTATTACAACAATCTTAGACAAATCACCACTATCAACTAGATTCCAGTTATCAATTCCATACAAACCATTGTTCCTGAATGTATATGGCTTAAAAAATATAATAGAATCAAGCTACCTTGGCCAGTCGCAGTGGCTACTCACATCTGTAATCCCAGCACTTTGGGAGAACAAGGCAAGAGGATCACTTGAGACCAGAAGTTCAAGACTAGCCTGGGCAACATAGTGAGACCCCTGTCCTTACAAAAAAATAAAAATAAAAAAATTAGCCTGGCATAGTGGCATGCACCTGTAGTCCTAGCTACTAGGGAGGCTGAGGTGGGAGAACCACTTGAGCACAGGCGATTGAGGCTGCAGTGGGCTATGATCACACCACTGTGCTCCAGTCTGGGCAACAGAAAAGAATCAAACTACCTTCTACTTTTAATGATTGTATTTTTGTTTTTTAAATATTTGAAATAAATTGATTCCAGTGTTTTCATGCATTGACTATTAATATCAATTTCTAGCCAAATTT

GGATTGCTACATAACTATACTTTTTTTTTTTTTTTTTTTTTGCAGTTTTGCCATTGCCACTAGCACAGACTTGTGATGATTAATTCTGGTGCTTTTTTTCCTTTTCTTCTCACAGATGTTGAAGTGCTGTTTTTTAAAGCTTTTCCCCCTATTTTGATGCATCAGATAAGGTACATTCTTGGAATGATGAACAACCCCTGACTTAAAATGTAACCAATATTGTGAAATTCCCCATCATTTTAAATTAAATACAAGCTGTTTTCAAGTCAGTCATTTTTAGATTATGGCTCTAAAAATAAATGATTCCCATGGCCTTAGTGCATTTTCTGCTCACACAAGTGAGCAATAAGGACAAATGGTTCCTTAGGATACAGTAAATACAGCAGAATGTATGTCAGTGTATCTCCATTATGTCTCATAAAAATGCTAAGTGGTATGTGTGTGTGCAGCCAAGTCTGCTACACAACTTGCGTAATACAGTGGTGATTTTACTAACCACACGGGAGGTTTCTTGGCCTTGGAAATAAGAATGGTTTTTTGTTGTTGCTGTTTTTAATATCATAAGCCATGAAAGCAATCTTCCATCAAATATTCAAATATAAACTGTTGACTTCCATTCAGACCTGCAAGCTGTATGGTGGACTTTGTAAGCTTCTTTTTGAAATTCGATGTTTCCAGGAAAGCACAAAAGGAGAAAGGAGGTACAATAAGGGGAAACAAAGTCACTGTTCCCCAGTTCTTTTTACTTGATCTTCTTCTTTTACTTGACTCTCCTTCCACTCCTTCAGCATAATCGGTTTTAATATCCGTAAGCATCTGGGGAGTCACCTGGGCTTTTAGTGACAAAAGTCTGTGGGCGAAGCCCCTCTGAGAAGAGAACTCTGCTCTCTAGGCCTTTCAGAGGTAGGCAGAGCTCGGCAACCGGTTTCTACGGACCGAGGACGCGTTGCTAAGGGACTGAAGGGTGGGGGCGGGGC

-- Factors predicted by PROMO in this sequence ----------------------

NAME; MATRIX_WIDTH;

CUTL1 [T00100]; **6**

-- PROMO predictions detail ------------------------------------------

Sequence name; Factor name; Start position; End position; Dissimilarity; String; RE equally; RE query

hg38_ncbiRefSeq_NM_001276352.2 range=chr1:67134971-67136970 5'pad=0 3'pad=0 strand=- repeatMasking=nÐ

; CUTL1 [T00100]; 56; 61; 0.967341; AGCAAT; 2.44141; 3.31524;

hg38_ncbiRefSeq_NM_001276352.2 range=chr1:67134971-67136970 5'pad=0 3'pad=0 strand=- repeatMasking=nÐ

; CUTL1 [T00100]; 164; 169; 2.026472; ATTGTA; 2.92969; 3.98721;

hg38_ncbiRefSeq_NM_001276352.2 range=chr1:67134971-67136970 5'pad=0 3'pad=0 strand=- repeatMasking=nÐ

; CUTL1 [T00100]; 419; 424; 1.467778; ATTGAC; 1.46484; 2.53052;

hg38_ncbiRefSeq_NM_001276352.2 range=chr1:67134971-67136970 5'pad=0 3'pad=0 strand=- repeatMasking=nÐ

; CUTL1 [T00100]; 515; 520; 0.925283; AACAAT; 2.44141; 3.31524;

hg38_ncbiRefSeq_NM_001276352.2 range=chr1:67134971-67136970 5'pad=0 3'pad=0 strand=- repeatMasking=nÐ

; CUTL1 [T00100]; 557; 562; 0.630875; **ATCAAT**; 0.48828; 1.17954;

hg38_ncbiRefSeq_NM_001276352.2 range=chr1:67134971-67136970 5'pad=0 3'pad=0 strand=- repeatMasking=nÐ

; CUTL1 [T00100]; 575; 580; 0.925283; ATTGTT; 2.44141; 3.31524;

hg38_ncbiRefSeq_NM_001276352.2 range=chr1:67134971-67136970 5'pad=0 3'pad=0 strand=- repeatMasking=nÐ

; CUTL1 [T00100]; 850; 855; 1.511826; ATTGAG; 1.46484; 2.53052;

hg38_ncbiRefSeq_NM_001276352.2 range=chr1:67134971-67136970 5'pad=0 3'pad=0 strand=- repeatMasking=nÐ

; CUTL1 [T00100]; 935; 940; 2.026472; ATTGTA; 2.92969; 3.98721;

hg38_ncbiRefSeq_NM_001276352.2 range=chr1:67134971-67136970 5'pad=0 3'pad=0 strand=- repeatMasking=nÐ

; CUTL1 [T00100]; 968; 973; 0.630875; ATTGAT; 0.48828; 1.17954;

hg38_ncbiRefSeq_NM_001276352.2 range=chr1:67134971-67136970 5'pad=0 3'pad=0 strand=- repeatMasking=nÐ

; CUTL1 [T00100]; 990; 995; 1.467778; ATTGAC; 1.46484; 2.53052;

hg38_ncbiRefSeq_NM_001276352.2 range=chr1:67134971-67136970 5'pad=0 3'pad=0 strand=- repeatMasking=nÐ

; CUTL1 [T00100]; 1003; 1008; 0.630875; **ATCAAT**; 0.48828; 1.17954;

hg38_ncbiRefSeq_NM_001276352.2 range=chr1:67134971-67136970 5'pad=0 3'pad=0 strand=- repeatMasking=nÐ

; CUTL1 [T00100]; 1025; 1030; 0.967341; ATTGCT; 2.44141; 3.31524;

hg38_ncbiRefSeq_NM_001276352.2 range=chr1:67134971-67136970 5'pad=0 3'pad=0 strand=- repeatMasking=nÐ

; CUTL1 [T00100]; 1075; 1080; 1.804245; ATTGCC; 2.92969; 3.98721;

hg38_ncbiRefSeq_NM_001276352.2 range=chr1:67134971-67136970 5'pad=0 3'pad=0 strand=- repeatMasking=nÐ

; CUTL1 [T00100]; 1235; 1240; 0.000000; ACCAAT; 0.48828; 0.67900;

hg38_ncbiRefSeq_NM_001276352.2 range=chr1:67134971-67136970 5'pad=0 3'pad=0 strand=- repeatMasking=nÐ

; CUTL1 [T00100]; 1241; 1246; 1.806234; ATTGTG; 2.92969; 3.98721;

hg38_ncbiRefSeq_NM_001276352.2 range=chr1:67134971-67136970 5'pad=0 3'pad=0 strand=- repeatMasking=nÐ

; CUTL1 [T00100]; 1364; 1369; 0.967341; AGCAAT; 2.44141; 3.31524;

hg38_ncbiRefSeq_NM_001276352.2 range=chr1:67134971-67136970 5'pad=0 3'pad=0 strand=- repeatMasking=nÐ

; CUTL1 [T00100]; 1595; 1600; 0.967341; AGCAAT; 2.44141; 3.31524;

hg38_ncbiRefSeq_NM_001276352.2 range=chr1:67134971-67136970 5'pad=0 3'pad=0 strand=- repeatMasking=nÐ

; CUTL1 [T00100]; 1725; 1730; 2.026472; TACAAT; 2.92969; 3.98721;

-- END ---------------------------------------------------------------
