# Supplementary material for: Overexpressed P75CUX1 promotes EMT in glioma infiltration by activating β-catenin
Source: Cell Death Dis. 2021 Feb 4;12(2):157. doi: 10.1038/s41419-021-03424-1 (PMC7862635; doi:10.1038/s41419-021-03424-1)
Supplement: Supplementary file 3 — Supplementary remark of CUX1 antibody [file 41419_2021_3424_MOESM3_ESM.docx]

**Sequence alignment of human CUX1-GST fusion protein using to make antibody and P75CUX1isoform：**

# Identity: 295/343 (86.0%)

# Similarity: 295/343 (86.0%)

# Gaps: 48/343 (14.0%)

# Score: 1374.0

#=======================================

EMBOSS_001 1 MAANVGSMFQYWKRFDLQQLQRELDATATVLANRQDESEQSRKRLIEQSR 50

||||||||||||||||||||||||||||||||||||||||||||||||||

EMBOSS_001 1 MAANVGSMFQYWKRFDLQQLQRELDATATVLANRQDESEQSRKRLIEQSR 50

EMBOSS_001 51 EFKKNTPEDLRKQVAPLLKSFQGEIDALSKRSKEAEAAFLNVYKRLIDVP 100

|||||||||||||||||||||||||||||||||||||||||||||||

EMBOSS_001 51 EFKKNTPEDLRKQVAPLLKSFQGEIDALSKRSKEAEAAFLNVYKRLI--- 97

EMBOSS_001 101 DPVPALDLGQQLQLKVQRLHDIETENQKLRETLEEYNKEFAEVKNQEVTI 150

| || ||||

EMBOSS_001 98 D-VP------------------------------------------EVTI 104

EMBOSS_001 151 KALKEKIREYEQTLKNQAETIALEKEQKLQNDFAEKERKLQETQMSTTSK 200

||||||||||||||||||||||||||||||||||||||||||||||||||

EMBOSS_001 105 KALKEKIREYEQTLKNQAETIALEKEQKLQNDFAEKERKLQETQMSTTSK 154

EMBOSS_001 201 LEEAEHKVQSLQTALEKTRTELFDLKTKYDEETTAKADEIEMIMTDLERA 250

||||||||||||||||||||||||||||||||||||||||||||||||||

EMBOSS_001 155 LEEAEHKVQSLQTALEKTRTELFDLKTKYDEETTAKADEIEMIMTDLERA 204

EMBOSS_001 251 NQRAEVAQREAETLREQLSSANHSLQLASQIQKAPDV--AIEVLTRSSLE 298

||||||||||||||||||||||||||||||||||||| |||||||||||

EMBOSS_001 205 NQRAEVAQREAETLREQLSSANHSLQLASQIQKAPDVEQAIEVLTRSSLE 254

EMBOSS_001 299 VELAAKEREIAQLVEDVQRLQASLTKLRENSASQISQLEQQLS 341

|||||||||||||||||||||||||||||||||||||||||||

EMBOSS_001 255 VELAAKEREIAQLVEDVQRLQASLTKLRENSASQISQLEQQLS 297

#---------------------------------------
