# Supplementary figures and images for: Overexpressed P75CUX1 promotes EMT in glioma infiltration by activating β-catenin
Source: Cell Death Dis. 2021 Feb 4;12(2):157. doi: 10.1038/s41419-021-03424-1 (PMC7862635; doi:10.1038/s41419-021-03424-1)

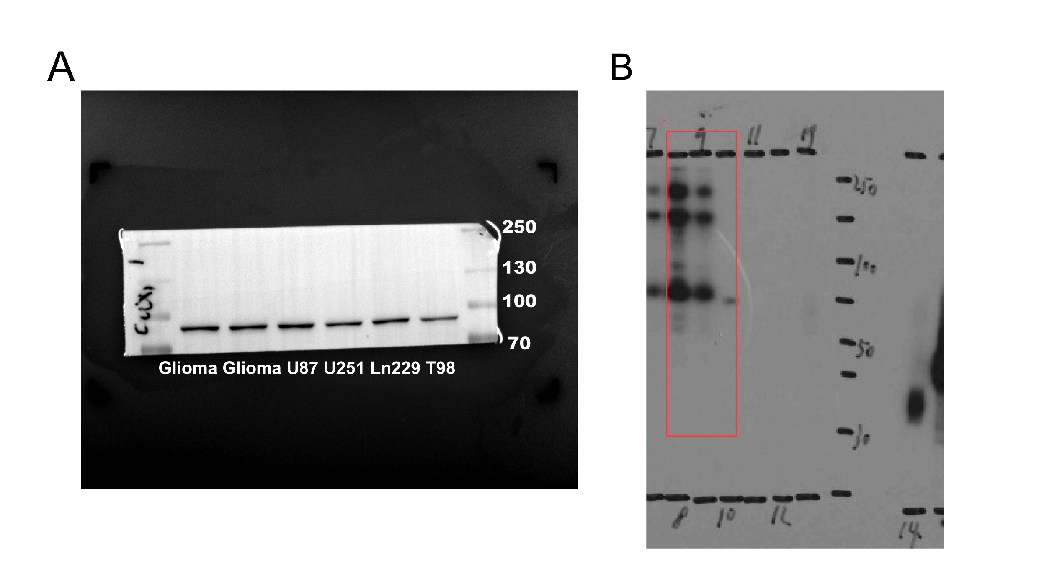

Supplement: Supplementary file 4 — Supplementary Fig. 1 [file 41419_2021_3424_MOESM4_ESM.tif]

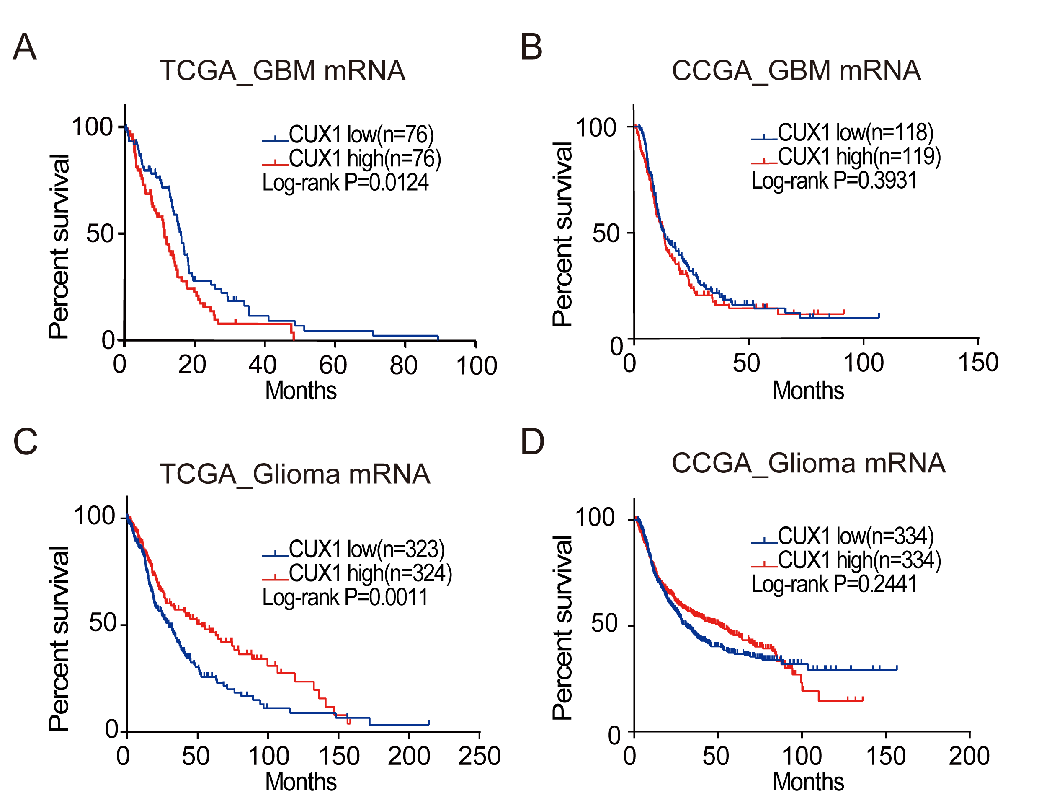

Supplement: Supplementary file 5 — Supplementary Fig. 2 [file 41419_2021_3424_MOESM5_ESM.tif]
